# Estimating marginal effects with zero-inflated models: A tutorial with the R package *mzim*

**DOI:** 10.3758/s13428-026-03036-7

**Published:** 2026-05-22

**Authors:** Chendong Li, Oi-Man Kwok, Timothy Lawrence

**Affiliations:** Department of Educational Psychology, Texas A&M University, Harrington Tower, College Station, TX 77843-4225 USA

**Keywords:** Count Data, Zero-inflation, Marginalized Zero-inflated Model, R Package

## Abstract

Count data in the psychological and health sciences are often characterized by an excess of zero values, a feature known as zero inflation. While traditional zero-inflated models, such as the zero-inflated Poisson (ZIP) and zero-inflated negative binomial (ZINB), were developed to handle such data, they present challenges for applied researchers. Standard count models can produce biased estimates, and the dual-parameter output of traditional zero-inflated models provides conditional effects for a latent-at-risk subpopulation, complicating interpretation and often failing to answer research questions about the entire population directly. To address these limitations, marginalized zero-inflated (mZI) models directly estimate the population-averaged effect, yielding a single, interpretable coefficient for each predictor’s overall effect. However, the adoption of mZI models has been hindered by the lack of an accessible software package. The current study has two objectives: first, it provides a tutorial on the theory, estimation, and interpretation of marginalized zero-inflated models. Second, it introduces *mzim*, a new R package designed to make both marginalized zero-inflated Poisson (mZIP) and Negative Binomial (mZINB) models readily accessible. Using an empirical example on self-reported youth abuse experiences, we demonstrate a complete workflow with the *mzim* package and compare the results from the mZINB model to traditional approaches, highlighting the practical benefits of the marginalized framework for applied researchers.

**Supplementary Information:**

The online version contains supplementary material available at 10.3758/s13428-026-03036-7.

## Overview

In psychological and medical research, count data—representing the number of times an event occurs—are frequently encountered (Agresti, 2002). Examples include the number of school absences per year or the number of chemotherapy sessions attended by cancer patients (Zorc et al., 2013; Chrischilles et al., 2009). Often, particularly when studying negative or infrequent behaviors in general populations, this data is characterized by a high number of zero-count observations, a phenomenon known as zero-inflation (ZI). Studies on substance use, violent behaviors, or risky sexual practices commonly yield such data, which is typically highly skewed (e.g., Bandyopadhyay et al., 2011; Swartout et al., 2015; Hu et al., 2011). These excess zeros surpass what would be expected if the data followed standard count distributions, reflecting a mixture of individuals who do not engage in the behavior and those who do, but may not have during the study period.

When confronted with zero-inflated count data, researchers have several analytical options, each with distinct advantages and limitations. One common approach to handling non-normal data is maximum likelihood estimation with robust standard errors (MLR), as implemented in software such as Mplus (Muthén & Muthén, 2017). This method uses a sandwich estimator to compute standard errors that are consistent even when assumptions are violated, such as heteroscedasticity. Its primary limitation, however, is that while MLR can correct for general non-normality, it is not designed to address the specific two-part data-generating process underlying zero-inflation. It fails to account for the mixture of structural and sampling zeros, and thus may not adequately model the phenomenon of interest.

Another approach is to ignore the zero-inflation and apply standard count models like Poisson or negative binomial (NB) regression. However, this misspecification leads to significant statistical issues. Numerous simulation studies have documented that applying standard count models to zero-inflated data can yield biased parameter estimates and, in some cases, an incorrect estimate of the effect direction (Lambert, 1992). Furthermore, the unaccounted-for variance from the excess zeros leads to underestimated standard errors, which results in an inflated type I error rate (Campbell, 2021).

To more accurately analyze data with excess zeros, researchers developed specialized models that explicitly account for the zero-inflation process (Preisser et al., 2012). The most widely used approaches are the zero-inflated Poisson (ZIP) model (Lambert, 1992) and the zero-inflated negative binomial (ZINB) model (Greene, 1994). These models explicitly accommodate the dual nature of zero-inflated data. The ZIP and ZINB models are two-part mixture models that conceptualize the data as arising from two distinct processes. The models combine a binary logistic model with a standard count model. The logistic component estimates the probability that an observation is a structural zero—originating from a subpopulation that has no chance of experiencing the event. The count component (Poisson for ZIP, negative binomial for ZINB) models the counts for the remaining subpopulation, which is considered at-risk of experiencing the event. For this at-risk group, a zero count is still possible and is referred to as a sampling zero. The ZINB model is an extension of the ZIP that adds a dispersion parameter to the count component, making it more flexible for data where the variance is greater than the mean (overdispersion).

These models are built on the assumption that zeros in the data arise from two distinct sources: structural zeros from individuals who will always be zero, and sampling zeros from individuals who could have a positive count but did not during the study period (He et al., 2014). For instance, in a study of cigarette use, zero counts include both participants who have never smoked (structural zeros) and smokers who simply did not smoke during the observation window (sampling zeros) (Pittman et al., 2022). Thus, the parameters from ZIP and ZINB models are interpreted within a latent class framework, distinguishing between the at-risk sampling zero subpopulation and not-at-risk structural zero subpopulations (Loeys et al., 2012). The coefficients from the logistic part describe how predictors relate to the odds of being in the structural zero group. The coefficients from the count part describe how predictors affect the outcome only for those within the at-risk group.

Despite their statistical elegance, traditional ZIP and ZINB models present significant challenges for applied researchers. The latent class interpretation often fails to align with the research objective. Researchers often want to assess the marginal effect of a predictor across the entire sample, rather than only a latent subgroup (Albert et al., 2014). For example, in an intervention study, the key question is often the average effect of the program on the total population, rather than the effect on the unobserved at-risk subgroup.

The dual-parameter output from ZIP and ZINB complicates analysis and communication. The coefficients from the count part do not represent the overall effect on the population mean. While methods exist to calculate this marginal effect post-estimation (e.g., using the delta method or bootstrapping), these techniques can be computationally intensive and difficult for applied researchers to implement correctly (Albert et al., 2014; Long et al., 2014).

The core assumption of two separated subpopulations (structural vs. at-risk) is often theoretically untestable due to the latent nature of the class membership. In applied research, the distinction between these zero types is frequently ambiguous, with no empirical method to differentiate them at the individual level. Consequently, imposing this rigid latent-class framework, which relies on these subpopulations for its primary interpretation, may fail to accurately reflect the true data-generating process. An alternative framework, the hurdle model, conceptualizes this process differently. Instead of assuming two types of zeros, hurdle models treat all zeros as a single group and model the data in two stages: a binary model determines whether an observation has a zero or a positive count, followed by a separate truncated count model for the positive values only. This approach avoids the distinction between structural and sampling zeros entirely. However, a detailed comparison of hurdle models and their own set of interpretations is beyond the scope of this paper.

To overcome these challenges, marginalized zero-inflated models (mZIP and mZINB) were developed as an accessible approach for estimating marginal effects (Long et al., 2014; Preisser et al., 2016). Instead of modeling two separate latent processes, these models are structured to directly model the population-averaged (marginal) mean count while still accounting for the excess zeros. The marginalized ZIP (mZIP) model directly links the overall mean count to covariates, allowing for straightforward inference on overall exposure effects. The interpretation becomes analogous to a standard Poisson regression but is corrected for the zero inflation. The marginalized ZINB (mZINB) model extends this framework by replacing the Poisson component with a negative binomial distribution, allowing it to handle both excess zeros and overdispersion simultaneously.

The marginalized approach directly resolves the primary challenges posed by traditional zero-inflated models. First, by directly modeling the marginal mean, the coefficients from an mZIP or mZINB model represent the effect of a predictor on the average outcome across the entire population. This provides a single, easy-to-interpret parameter that directly answers the most common type of research question about overall effects.

Second, marginalized models provide parameter estimates that have the same straightforward interpretation as those from standard Poisson or NB regression (i.e., log-incidence density ratios). This eliminates the need for complex post-estimation calculations to derive marginal effects, making the results more accessible and less prone to misinterpretation.

Lastly, a key conceptual advantage of the marginalized approach lies in its handling of the untestable assumption regarding the origin of zero counts. It is important to note that marginalized models share the exact same structural assumption about the data-generating process, including the use of a logistic component. However, they shift the analytical focus. In a traditional ZIP or ZINB model, the logistic component is central to the interpretation, defining latent classes (at-risk vs. not-at-risk) that are necessary to understanding the count parameters. In contrast, the primary role of the logistic component in a marginalized model is statistical rather than interpretive. It functions as a mathematical mechanism to adjust for the distributional properties of the data—namely, the excess zeros—thereby enabling an unbiased and accurate estimation of the marginal effect. Consequently, the marginalized framework implies that while the dual-source nature of zeros (structural vs. sampling) may exist as the underlying data-generating mechanism, this latent separation becomes subordinate to the estimation of the overall mean. The logic of the mZI approach is not that it assumes the absence of the existence of structural zeros. However, it structures the inference in a way that distinguishing between the two sources is no longer required to interpret the overall exposure effect. By focusing the count component on the marginal mean, the separation of subpopulations, even if theoretically present, becomes irrelevant for answering the research question regarding the population-averaged effect.

The implementation of traditional ZIP and ZINB models for applied researchers has been facilitated by several statistical software packages, including the *pscl* (Zeileis et al., 2008) and *glmmTMB* (McGillycuddy et al., 2025) packages in R (R Core Team, 2024), the *zip* and *zinb* commands in Stata (StataCorp, 2023), and PROC GENMOD in SAS (SAS Institute Inc., 2022). In contrast, the implementation of the marginalized versions has been more limited, creating a gap between methodological development and practical application. In R, for instance, the mZIP model can be estimated using packages such as mcount and mzipmed. However, a limitation is the absence of a single, unified package that offers estimation for both the mZIP model and its overdispersed counterpart, the mZINB model. Accessible implementations for marginalized zero-inflated models are not readily available in other widely used statistical programs like SAS or Stata. This presents a barrier for applied researchers wishing to adopt these more interpretable models.

The current study has two objectives. First, this paper serves as a tutorial on the formulation, estimation, and implementation of mZIP and mZINB models. We present the underlying statistical theory of these models. Second, we introduce the *mzim* (Li, 2026), a newly developed R package designed to make them readily accessible to applied researchers. The major advantage of this package is that it can compute marginal estimates based on the entire sample, rather than a subsample, as in the traditional ZIP and ZINB models, when the data contain sampling zeros or a mix of both structural and sampling zeros. To demonstrate the complete workflow, from model specification to the interpretation of results, we analyze an empirical dataset using the *mzim* package. The paper will be organized as follows: we will detail the derivations for both the regular ZIP and ZINB models, as well as their marginalized versions. Then, we will introduce the dataset we are using to illustrate the proposed methods. Next, we will present the workflow of using mZIP/mZINB models in the empirical dataset. Lastly, we will compare and interpret the results from both analyses.

## Statistical model

### ZIP and ZINB models

The traditional ZIP models for handling zero-inflated count data were first introduced by Lambert (1992), assuming that the count variable. ${Y}_{i}$ for the $i$ th observation arises from two distinct processes: (1) a zero-inflation process with probability ${\psi}_{i}$ - the observation is an excess zero, representing a structural zero that cannot take on positive count values, and (2) count process with probability $1-{\psi}_{i}$. The count process $g\left({y}_{i}|{\theta}_{i}\right)$ follows a standard Poisson distribution with mean ${{\theta}_{i}=\mu }_{i}$, allowing for both zero and positive counts. Thus, the probability distribution of ${Y}_{i}$ in the ZIP model is defined as follows

1 $$\begin{array}{c}P\left({Y}_{i}=0\right)={\psi}_{i}+\left(1-{\psi}_{i}\right){e}^{{-\mu }_{i}}\\ P\left({Y}_{i}={y}_{i}\right)=\left(1-{\psi}_{i}\right)g\left({y}_{i}|{\theta}_{i}\right), {y}_{i}\in {\mathbb{Z}}^{+}\end{array}$$

When the count process exhibits overdispersion, meaning the sample variance is greater than the sample mean, it violates the Poisson distribution assumption. The count process will instead follow a NB distribution with ${{\theta}_{i}=(\mu }_{i};\phi )$ where $\phi$ is the overdispersion parameter, which is referred as ZINB (Greene, 1994). This allows the variance to be specified as a quadratic function of the mean in the count process: $Var\left({y}_{i}\right)={\mu}_{i}+\phi {\mu}_{i}^{2}$.

To assess the impact of covariates on the count distribution in a ZIP or ZINB model, two processes could be explicitly expressed as a function of covariates. The most natural choice to model the probability of excess zeros is to use a logistic regression model,

2 $$logit({\psi}_{i}) = {Z}_{i}^{\prime}\lambda$$

where ${Z}_{i}$ is a vector of covariates influencing the structural zero process and $\lambda$ is a vector of parameters associated with ${Z}_{i}$. Other link functions (e.g., probit link) can be used as well, but will not be considered here. Each element ${\lambda}_{j}$ represents the log-odds change in the probability of an observation being a structural zero for a one-unit increase in the $j$ th covariate in ${Z}_{i}$. A positive ${\lambda}_{j}$ indicates that as the $j$ th covariate in ${Z}_{i}$, the odds of the observation being a structural zero increase.

For the count process, the impact of covariates excluding the excessive zeros can be modeled through Poisson regression or NB regression below

3 $$\mathrm{log}\left({\mu}_{i}\right)={X}_{i}^{\prime}\beta$$

where ${X}_{i}$ is a vector of covariates influencing the count process and $\beta$ is a vector of parameters associated with ${X}_{i}$. Each element ${\beta}_{j}$ represents the log change in the expected count ${\mu}_{i}$ for a one-unit increase in the $j$ th covariate in ${X}_{i}$, among observations not in the excess zero group. Exponentiating $\beta$ yields the incidence rate ratio (IRR), indicating the multiplicative effect on the expected count. In practice, models often have specification either ${Z}_{i}={X}_{i}$ or that ${Z}_{i}$ consists of a subset of the covariates ${X}_{i}$ in the count process (Preisser et al., 2016)

It is important to note that in the traditional ZIP and ZINB models, researchers should be cautious when interpreting the parameters $\lambda$ and $\beta$ separately within their respective processes. Specifically, each element ${\lambda}_{j}$​​ in the zero-inflation component represents the log-odds ratio of a one-unit increase in the $j$ th covariate in ${Z}_{i}$​ on the probability of an observation being an excess zero. Similarly, each element ${\beta}_{j}$​ in the count component represents the log-incidence rate ratio (L-IRR) of a one-unit increase in the $j$ th covariate in ${X}_{i}$​ on the mean count ${\mu}_{i}$ among individuals not in the excess zero group. However, researchers are often more interested in the overall effect of predictors on the entire population mean of the outcome rather than effects confined to latent subpopulations. This presents a challenge in the traditional ZIP and ZINB models because there is no simple summary of the effect of a one-unit increase in a predictor on the overall population mean ${\nu}_{i}=E({Y}_{i})$, which is frequently the primary focus of investigation. The marginal mean of the outcome ${\nu}_{i}$ is given by

4 $$E\left(Y\right)={\nu}_{i}= \left(1-{\psi}_{i}\right){\mu}_{i}=\frac{{e}^{{X}_{i}^{\prime}\beta }}{1+{e}^{{Z}_{i}^{\prime}\lambda }}$$

indicating that the overall mean depends on both the zero-inflation parameters $\lambda$ and the count parameters $\beta$. This dependency between predictors and the marginal mean is complex and nonlinear, making it difficult to directly interpret the impact of a one-unit increase in a predictor on the overall outcome within the traditional ZIP model.

## Marginalized ZI models

To address the challenges of interpreting the overall effect of predictors on the entire population mean within the traditional ZIP and ZINB models, the mZIP and mZINB models were developed (Long et al., 2014; Preisser et al., 2016). These marginalized models use a similar framework that modifies the traditional approaches by directly modeling the marginal mean of the outcome variable, providing a more straightforward interpretation of predictor effects on the overall population.

In the mZIP model, we begin by specifying the zero-inflation process similarly to the traditional ZIP model. The probability of observation being an excess zero is modeled using a logistic regression as before in Eq. (2) and solving for ${\psi}_{i}$ using the inverse logit function, we obtain

5 $${\psi}_{i}=\frac{1}{1+{e}^{{Z}_{i}^{\prime}\lambda }}$$

Next, instead of modeling the count process conditional on the outcome not being in the latent class of excess zeros, the mZIP model directly models the marginal mean ${\nu}_{i}$ of the outcome for the entire population using a log-linear relationship

6 $$\mathrm{log}\left({\nu}_{i}\right)={X}_{i}^{\prime}\alpha$$

where ${\nu}_{i}=E({Y}_{i})$ is the expected count for the $i$ th observation, ${X}_{i}$ is a vector of covariates associating with the overall mean, and $\alpha$ is a vector of parameters associated with ${X}_{i}$. Exponentiating both sides yields

7 $${\nu}_{i}={e}^{{X}_{i}^{\prime}\alpha }$$

This formulation allows the parameters $\alpha$ to be interpreted similarly to those in standard Poisson regression, providing a direct interpretation of the effect of predictors on the marginal mean ${\nu}_{i}$. As previously given in Eq. (4) ${\nu}_{i}= \left(1-{\psi}_{i}\right){\mu}_{i}$, with the known functional form of ${\nu}_{i}$, we could solve for ${\mu}_{i}$, the mean of the count process in the traditional zero-inflated Poisson and substituting the expressions for ${\nu}_{i}$ in Eq. (7) and ${\psi}_{i}$ in Eq. (5) into the equation for ${\mu}_{i}$, we get

8 $${\mu}_{i}=\frac{{\nu}_{i}}{1-{\psi}_{i}}=\frac{{e}^{{X}_{i}^{\prime}\alpha }}{1-\left(\frac{{e}^{{Z}_{i}^{\prime}\lambda }}{1+{e}^{{Z}_{i}^{\prime}\lambda }}\right)}={e}^{{X}_{i}^{\prime}\alpha }\left(1+{e}^{{Z}_{i}^{\prime}\lambda }\right)$$

In order to use the ZIP model likelihood framework, we redefine ${\mu}_{i}={e}^{{\delta}_{i}}$ and take the natural logarithm of both sides. Here, ${\delta}_{i}$ is not necessarily a linear function of model parameters, highlighting the interpretational challenges inherent in traditional ZIP models

9 $${\delta}_{i}=\mathrm{log}{\mu}_{i}={X}_{i}^{\prime}\alpha +\mathrm{log}(1+{e}^{{Z}_{i}^{\prime}\lambda })$$

By substituting Eq. (5) and Eq. (6) into Eq. (9), the likelihood of the mZIP model for $(\lambda , \alpha )$ is

10 $$\begin{array}{c}L\left(\lambda , \alpha |y\right)= \prod\limits_{{y}_{i}}{\left(1+{e}^{ {Z}_{i}^{\prime}\lambda }\right)}^{-1}\prod\limits_{{y}_{i}=0}{(e}^{ {Z}_{i}^{\prime}\lambda }+{e}^{-(1+\mathrm{exp}{(Z}_{i}^{\prime}\lambda ))\mathrm{e}\mathrm{x}\mathrm{p}({X}_{i}^{\prime}\alpha )})\\ \times \prod_{{y}_{i}>0}[{e}^{-(1+\mathrm{exp}{(Z}_{i}^{\prime}\lambda ))\mathrm{e}\mathrm{x}\mathrm{p}({X}_{i}^{\prime}\alpha )}{\left(1+{e}^{{Z}_{i}^{\prime}\lambda }\right)}^{{y}_{i}}{e}^{{X}_{i}^{\prime}\alpha {y}_{i}}/({y}_{i}!)]\end{array}$$

For datasets where the variance of the count data is greater than the mean, the mZIP model can be extended to the mZINB model. The mZINB model provides an additional overdispersion parameter by using a NB distribution for the count process, while keeping the population-averaged interpretation of the marginalized framework. The mZINB model retains the same direct modeling approach for the zero-inflation probability ${\psi}_{i}$, and the marginal mean ${\nu}_{i}$​, as defined previously. The probability of an excess zero ${\psi}_{i}$ is specified using the logistic model in Eq.~(5), and the marginal mean ${\nu}_{i}$ is modeled with the log-linear relationship shown in Eqs.~(6) and (7). The relationship used to solve for the mean of the count process ${\mu}_{i}$ remains the same in Eq.~(8) but the distribution assumed for these counts is NB rather than Poisson. The core of the mZINB model is its use of the negative binomial (NB) distribution, whose probability mass function is detailed in the Appendix. The NB distribution is defined by its expected mean $E\left({y}_{i}\right)={\mu}_{i}$ and a variance function of $Var\left({y}_{i}\right)={\mu}_{i}+\phi {\mu}_{i}^{2}$. The overdispersion parameter $\phi$ allows the variance to exceed the mean. The likelihood for the mZINB model is therefore constructed by substituting this NB probability function for the Poisson in the marginalized framework in Eq.~(10). The resulting log-likelihood function is shown in the Appendix.

For the mZIP and mZINB, to facilitate their application, we have developed *mzim*, an R package that uses the quasi-Newton BGFS algorithm to perform the estimation. The function generates robust starting values from a standard ZIP or ZINB fit. The package provides both model-based standard errors derived from the Hessian matrix and robust sandwich estimators for variance estimation. In the following session, we will use an empirical example to illustrate the package’s use.

## Empirical example and R illustration

**Fig. 1:**
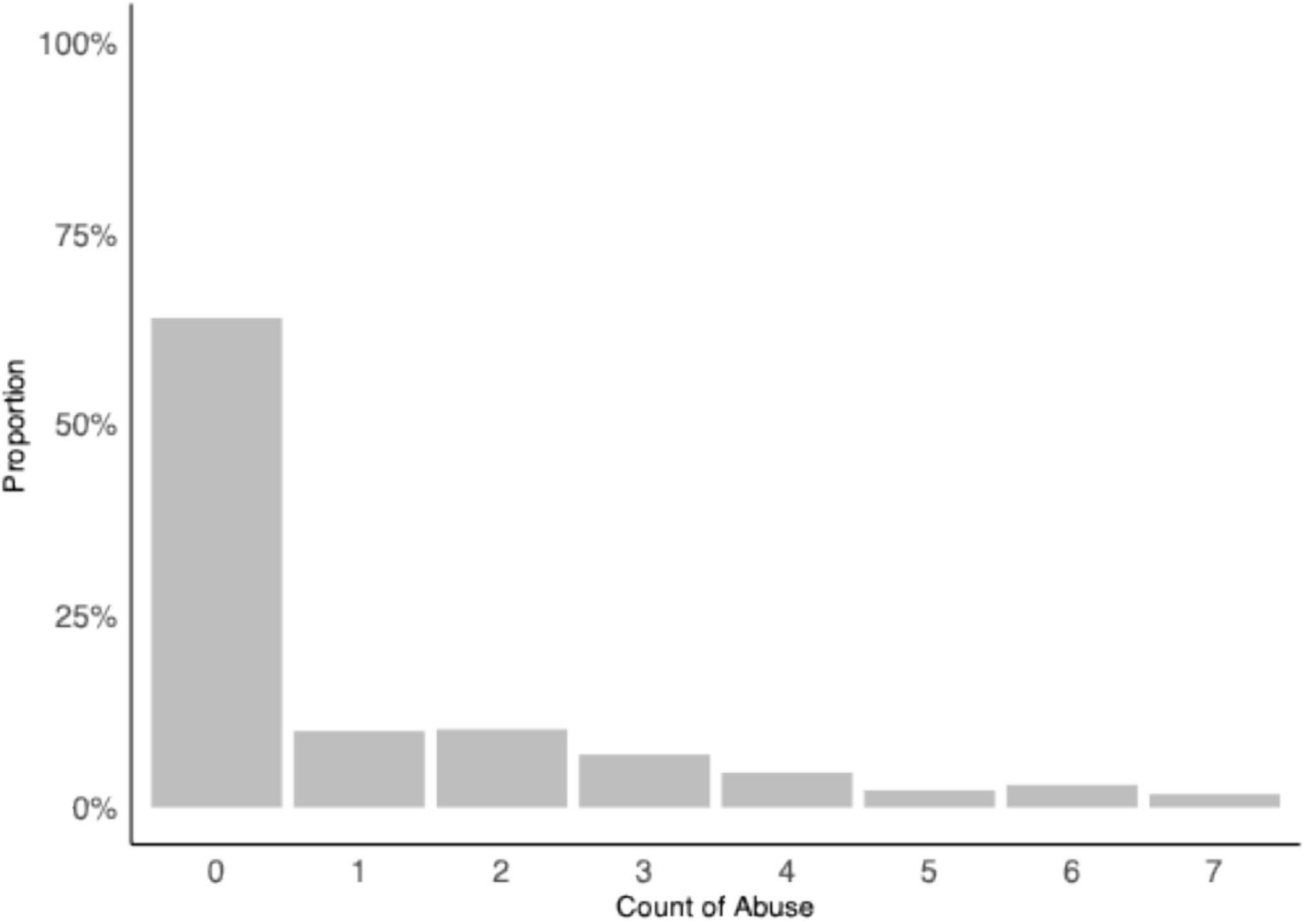
Histogram of abusive event counts (*N* = 424)

To illustrate our modeling approaches, we use data from the study *Crime during the transition to adulthood: how youth fare as they leave out-of-home care in Illinois, Iowa, and Wisconsin, 2002–2007* (Courtney & Cusick, 2010). This longitudinal research’s aim was to examine how supports and services provided by these states—under varying child welfare policies—affected youth outcomes such as education, employment, housing stability, and crime. Youth who had been in care for at least one year prior to age 17 and placed due to abuse or neglect were followed over multiple interview waves (e.g., at ages 17–18, 19–20, and 21+). For the present empirical example, we focus on Wave 1 data to examine abuse experiences among 424 participants with complete information. Specifically, our outcome of interest is number of abusive events reported during Wave 1 interviews. Figure 1 shows the distribution of abusive event counts, which contains 270 zeros (64%) and 154 non-zeros (36%).

This demonstration investigates the association between an individual’s gender and school enrolment status and their self-reported number of abuse experiences at Wave 1. The primary predictors are gender (0 = female, 1 = male) and school enrolment, a dichotomous variable where a value of 1 indicates that the youth was enrolled in any form of school (high school, vocational, or college) and 0 indicates otherwise. Table 1 shows the counts of youth with zero or one or more abusive experiences broken down by their gender and school enrollment status.

**Table 1:** Distribution of participants by abuse experience, gender, and school enrollment status (*N* = 424)

		Abuse counts	
Gender	School enrollment	Zero events	One or more events
Female	Enrolled	70	39
	Not enrolled	81	38
Male	Enrolled	63	33
	Not enrolled	56	44
Total		270	154

## Test for overdispersion

 Before fitting a zero-inflated model, it is crucial to select the appropriate underlying distribution for the count process. It requires a test for overdispersion, a condition in which the variance of the count data exceeds its mean, thereby violating a key assumption of the Poisson distribution. The NB distribution serves as an alternative, as it includes an additional parameter to account for this excess variability, providing a better fit in such situations. To statistically evaluate the presence of overdispersion, a likelihood-ratio test (LRT) is performed to compare the fit of a standard Poisson model with that of an NB model (Cameron & Trivedi, 2013). For a valid comparison, both models must be specified with the identical set of covariates. The null hypothesis (${H}_{0}$​) of the LRT is that no overdispersion exists, implying the Poisson model is sufficient. The alternative hypothesis (${H}_{1}$​) states that the data are significantly overdispersed, indicating the NB model offers a better fit. In R, this test can be implemented using the *odTest()* function from the *pscl* package (Zeileis et al., 2008). A statistically significant result (${\chi }^{2}=261.10$, *p* value < 0.01) from this test with race and school enrolment as the covariates provides the justification for selecting an NB-based model, such as ZINB or mZINB, for the subsequent analysis.

## Test for zero inflation

It is also important to test for the presence of zero inflation, a condition where the observed zero counts exceed what is expected under a standard distribution. To statistically evaluate the presence of zero-inflation and complement the visual inspection of the histogram, a Vuong test (Vuong, 1989) is performed to compare the fit of a standard NB model with that of a traditional ZINB model. Because these models are non-nested, the Vuong test utilizes a likelihood-ratio-based statistic. The null hypothesis (${H}_{0}$) of the Vuong test is that the two models are indistinguishable (i.e., equally close to the true data-generating process), implying the standard NB model is sufficient. The alternative hypothesis (${H}_{1}$) states that one model (i.e., the zero-inflated model) provides a strictly superior fit. In R, this test can be implemented using the *vuong()* function from the *pscl* package (Zeileis et al., 2008). A statistically significant result (*z* = – 3.70, *p* < 0.001; AIC-corrected *z* = – 3.14, *p* < 0.001; BIC-corrected *z* = – 2.00, *p* = 0.023) from this test with gender and school enrollment as the covariates provides empirical justification for selecting a zero-inflated framework for the subsequent analysis.

## Results

In order to demonstrate the application of the *mzim* package and compare the mZINB model against other common approaches, we analyze the predictors of self-reported abuse experiences using the Wave 1 data. We will compare three distinct methods: MLR, the traditional ZINB model, and the mZINB model.

First, we fit a linear regression model using MLR. Despite the outcome being count data, applied researchers might default to this approach due to its familiarity and the availability of robust standard error corrections (MLR) in statistical software (e.g., Mplus), which are often seen as a simple solution for violations of normality and homoscedasticity. The linear model is specified as:

11 $${Y}_{i}={\delta}_{0}+{\delta}_{1}Gende{r}_{i}+{\delta}_{2}Schoo{l}_{i}+{\epsilon}_{i}$$

where ${Y}_{i}$ is the abusive events for individual $i$, $Gende{r}_{i}$ is a dichotomous variable for the $i$ th individual (1 = male, 0 = female), and $Schoo{l}_{i}$ is the $i$ th individual’s school enrollment status (1 = enrolled, 0 = not enrolled). The results in Table 2 show that being male was significantly associated with a decrease of 0.39 abusive events on average compared to females (${\delta}_{1}= -0.39, p$ value = 0.014). School enrollment was not found to be a statistically significant predictor (${\delta}_{2}= 0.247, p$ value = 0.256). Interpreting these coefficients presents fundamental challenges stemming from model misspecification. First, the model imposes an additive structure on the data, which is conceptually misaligned with count outcomes where predictor effects are typically understood to be multiplicative (e.g., as incidence rate ratios). Second, the linear model fails to account for the data-generating process inherent to zero inflation.

**Table 2:** ZINB and mZINB results for the number of self-reported abuse events

	MLR				ZINB				mZINB			
Variable	Coefficient	Estimate	S.E.	*p* value	Coefficient	Estimate	S.E.	*p* value	Coefficient	Estimate	S.E.	*p* value
					*Structural zero process*							
Intercept	${\delta}_{0}$	0.976	0.203	< 0.001	${\gamma}_{0}$	– 0.336	0.088	< 0.001	${\gamma}_{0}$	0.928	0.286	0.001
Gender	${\delta}_{1}$	– 0.392	0.160	0.014	${\gamma}_{1}$	0.046	0.095	0.623	${\gamma}_{1}$	0.213	0.238	0.371
School enrollment	${\delta}_{2}$	0.247	0.217	0.256	${\gamma}_{2}$	– 0.289	0.098	0.003	${\gamma}_{2}$	– 0.832	0.306	0.007
					*Count process*							
Intercept					${\beta}_{0}$	1.261	0.177	< 0.001	${\alpha}_{0}$	– 0.017	0.248	0.977
Gender					${\beta}_{1}$	– 0.320	0.142	0.024	${\alpha}_{1}$	– 0.425	0.166	0.032
School enrollment					${\beta}_{2}$	– 0.306	0.183	0.094	${\alpha}_{2}$	0.228	0.257	0.375
Overdispersion					$\phi$	6.740	4.158		$\phi$	6.846	4.243	

Next, we fit the traditional ZINB model shown in Eq.~(12)

12 $$\begin{array}{c}logit\left({\pi}_{i}\right)= {\gamma}_{0}+ {\gamma}_{1}Gende{r}_{i}+{\gamma}_{2}Schoo{l}_{i}\\ \mathrm{log}\left({\mu}_{i}\right)={\beta}_{0}+ {\beta}_{1}Gende{r}_{i}+{\beta}_{2}Schoo{l}_{i}\end{array}$$

The same set of covariates is both included in the count and structural zero process in the ZINB model. Parameter estimation obtained from *zeroinfl()* function from package *pscl* in R is shown in Table 2. First, the ZI process models the likelihood of an individual belonging to the structural zero or never-abused group. In this component, gender was not a significant predictor of group membership (${\gamma}_{1}$ = 0.046, *p* value =.623). However, school enrollment was highly significant (${\gamma}_{2}$ = − 0.289​, *p* value =.003). The odds ratio of 0.749 (exp(− 0.289)) indicates that the odds of belonging to the structural zero group were 25.1% lower for youth enrolled in school compared to those not enrolled. Second, the count process models the expected number of abuse incidents for the subpopulation of youth that already considered at-risk. Within this group, being male was significantly associated with a 27.4% lower expected abuse count compared to females (IRR = 0.73; ​${\beta}_{1}$ = − 0.320, *p* value =.024). Being enrolled in school was not statistically significantly associated with the expected abuse count (${\beta}_{2}$ ​= − 0.306, *p* value =.094). Despite its statistical appropriateness, the traditional ZINB model presents the exact real-world challenges for applied researchers discussed earlier. The primary research question—What is the overall effect of gender and school enrollment on abuse for all youth?—is not directly answered. Instead, the model provides two separate answers for unobserved subgroups. This difficulty arises from the model’s foundational and non-testable assumption about the nature of the zero counts. The ZINB framework posits that the observed zeros are a mix of two types: structural zeros from youth who are truly not at risk and would never be abused, and sampling zeros from youth who are at risk but happened to not experience an abusive event during the data collection window. However, this categorization is purely theoretical. For any given youth with a zero count, it is impossible to empirically verify which group they belong to. The statistical inference of the model, which produces separate estimates for the at-risk and never-abused groups, depends on the validity of this untestable assumption.

Lastly, we fit the mZINB model using the same set of covariates, with the specification detailed in Eq.~(13).

13 $$\begin{array}{c}logit\left({\pi}_{i}\right)= {\gamma}_{0}+ {\gamma}_{1}Gende{r}_{i}+{\gamma}_{2}Schoo{l}_{i}\\ \mathrm{log}\left({\nu}_{i}\right)={\alpha}_{0}+ {\alpha}_{1}Gende{r}_{i}+{\alpha}_{2}Schoo{l}_{i}\end{array}$$

In contrast to the traditional ZINB, which models the conditional mean (${\mu}_{i}$) for the non-zero subgroup, the mZINB directly models the marginal mean ($\nu =E({Y}_{i})$) for the entire population as a function of gender and school enrollment. The logistic model for the structural zero process remains identical to that of the traditional ZINB. The R code implementing this model with the *mzim()* function in *mzim* package is shown below. Its key arguments, *count_formula* and *zi_formula*, specify the models for the marginal mean and structural zero process, respectively. Additionally, while the package computes robust sandwich estimators by default, model-based standard errors derived from the Hessian matrix can be requested by setting *robust = FALSE* in the summary function, as demonstrated. The resulting parameter estimates are shown in Table 2.
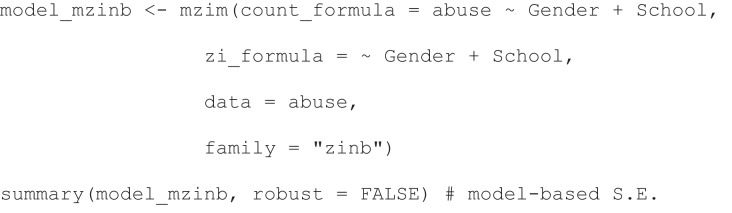


The zero-inflation process in the mZINB, which models the odds of belonging to the structural zero or never-abused group, is interpreted identically to the traditional ZINB. In this component, gender was not a significant predictor of group membership (${\gamma}_{1}$ ​= 0.213, *p* value =.371), but school enrollment was significant (${\gamma}_{2}$ ​= − 0.832, *p* value =.007). The odds ratio is 0.435 (exp(− 0.832)), indicating that the odds of being a structural zero were 43.5% lower for youth in school. In other words, individuals enrolled in school were less likely to report and experience abusive events. The key distinction between this model and the traditional ZINB lies in the marginal mean process. Unlike the traditional ZINB which estimates effects conditional on the at-risk subpopulation, this component models the overall average abuse count for the entire population. For this marginal mean, male participants were significantly associated with a 34.6% lower expected abuse count (or reported fewer abusive events) across the whole sample compared to their female counterparts (IRR = 0.65; ${\alpha}_{1}$​= − 0.425, *p* value =.032). School enrollment was not significantly associated with the overall average abuse count (${\alpha}_{2}$ ​= 0.228, *p* value =.375). The mZINB model’s results resolve the interpretive challenges posed by the preceding analyses. The advantage is that the marginal mean component provides a direct answer to the research question regarding population-level effects. For instance, the model yields a single incidence rate ratio (IRR = 0.71) for gender, representing its overall effect across the entire sample. This direct inference that was unavailable from the traditional ZINB model. Also, the role of the zero-inflation component is reconceptualized. Rather than defining interpretive latent classes, it serves as a nuisance parameter(s) that accounts for the excess zeros, allowing the marginal effect to be estimated without bias. The validity of the main finding is therefore no longer dependent on the untestable assumption about the nature of the zeros.

## Discussion

This paper has served as a tutorial on the formulation, estimation, and implementation of marginalized zero-inflated models. We have presented the underlying statistical theory for both traditional and marginalized versions of the ZIP and ZINB models and demonstrated their application using an empirical example. Another contribution of this work is the introduction of the *mzim* R package, a user-friendly tool to make mZIP and mZINB models readily accessible to applied researchers in psychology, health, and other social sciences. By providing straightforward implementation, we bridge the gap between complex statistical theory and practical data analysis.

Using an empirical example, the advantage of the marginalized approach becomes evident when contrasted with the other models. The linear model with MLR, while simple to implement, was misspecified. It imposed an additive structure on a multiplicative process and failed to account for the two-part data generating mechanism of zero-inflation, making its coefficient difficult to interpret meaningfully. The traditional ZI models also presented the core interpretive challenge, which this paper sought to address. It produces two distinct results for each predictor: one for the odds of belonging to the structural zero group, and another for the outcome count only within the latent at-risk subpopulation. While statistically sound, this fails to directly answer the researcher’s question, if it is about the overall effect of a predictor on the entire population. The interpretation is conditional on an assumption about the nature of zeros for each participant. The marginalized model, on the other hand, provides a single, interpretable coefficient for each predictor that represents the population-averaged (marginal) effect. In this framework, the zero-inflation component is reconceptualized not as a tool for interpreting latent classes, but as a statistical adjustment (i.e., a nuisance parameter) that corrects for the data’s distributional properties.

A central goal of this tutorial is to clarify for applied researchers the differences between the two frameworks. A theoretical distinction between the two modeling frameworks lies in their conceptualization of the zero values observed in the data. Traditional zero-inflated models (ZIP and ZINB) are essentially mixture models that differentiate zeros into two latent categories: structural zeros and sampling zeros. Under this framework, structural zeros are viewed as originating from a subpopulation that is immune to the event—individuals who are not at-risk and therefore have a probability of zero for the outcome. Conversely, sampling zeros are considered to arise from the count distribution itself; these represent individuals who are at-risk and capable of experiencing the event but simply did not do so during the specific observation window. Valid inference in traditional models relies on the assumption that this latent structure accurately reflects the true data-generating process.

However, the marginalized framework (mZIP and mZINB) shifts the inferential focus. The logic of using mZI models is not based on the assumption that structural zeros do not exist, but rather that distinguishing them from sampling zeros is unnecessary for marginal inference. The model statistically accounts for the excess zeros through the logistic component (i.e., acknowledging the distributional reality of the zero inflation), but parameterizes the count component to marginalize over these latent classes. Thus, while the separation of zeros into at-risk and not-at-risk groups may theoretically exist, the marginalized estimator treats this latent structure as a nuisance characteristic rather than a primary target of inference, allowing for a direct interpretation of effects across the entire population.

The choice between the two frameworks is also not merely technical but is dictated by the research question. Traditional ZIP/ZINB models are appropriate when the goal is to understand latent heterogeneity, which is to separately model the predictors of belonging to a not-at-risk group versus the predictors of event frequency among the at-risk subpopulation. In contrast, marginalized mZIP/mZINB models are the superior choice when the research question concerns the population-averaged impact of a predictor across an entire sample, a common scenario in psychological science. Selecting the marginalized model provides a direct answer to this type of question and avoids the common errors that misinterpret the conditional coefficients from a traditional ZINB model as marginal effects (Preisser et al., 2016). Another contribution of this work is bridging the gap between this advanced statistical method and its practical application. The *mzim* R package provides a user-friendly tool to make both mZIP and mZINB models readily accessible, allowing applied researchers to focus on answering their research questions correctly rather than on complex post-estimation calculations.

While this tutorial focuses on independent observations, marginalized zero-inflated models can be extended to accommodate the longitudinal structures often encountered in psychological research, such as the multiple interview waves in our empirical example. Long et al. (2015) extended the mZIP framework to include random effects, allowing researchers to account for within-subject correlation in repeated measures data while retaining the population-averaged interpretation of the marginal mean. However, estimating these mixed-effects marginalized models via traditional frequentist methods presents practical limitations. They are highly computationally intensive, often requiring complex numerical integration (e.g., adaptive Gauss-Hermite quadrature), and are prone to convergence issues, particularly with smaller sample sizes. Recent methodological work has begun addressing these barriers. For example, Li and Luo (2026) introduced a Bayesian marginalized zero-inflated Poisson model with random effects, demonstrating that a Bayesian framework can overcome frequentist convergence failures and provide robust marginal inference for longitudinal count data even within the small-sample context of single-case experimental designs. While the *mzim* package currently focuses on cross-sectional data to provide an accessible statistical tool, future development will expand these marginalized frameworks by incorporating both mixed-effects and Bayesian estimation to handle clustered and longitudinal data.

In summary, this paper seeks to encourage the use of marginalized zero-inflated models. By moving beyond the restrictive latent class interpretations of traditional models, researchers can now address questions about population-level effects with greater clarity and statistical rigor. It is our hope that the theoretical explication and the practical tools provided via the *mzim* package will encourage the broader adoption of these methods, ultimately leading to more accurate inference in psychological and health research. 

## Electronic supplementary material

Below is the link to the electronic supplementary material.

Supplementary file1 (CSV 5 KB)

## Data Availability

The code and data used in the demonstration are available at the following OSF repository.

## Appendix

### Marginalized zero-inflated negative binomial (mZINB) likelihood

The likelihood function for the mZINB model is constructed by replacing the Poisson distribution in the marginalized framework with a Negative Binomial (NB) distribution. We begin with the standard probability mass function (PMF) for a ZINB mixture distribution, which accounts for both the structural zero process (${\psi}_{i}$) and the NB count process:

$$P({Y}_{i}={y}_{i})=\left\{\begin{array}{cc}{\psi}_{i}+(1-{\psi}_{i}){P}_{NB}({Y}_{i}=0)& if \,{y}_{i}=0\\ \left(1-{\psi}_{i}){P}_{NB}({Y}_{i}={y}_{i}\right)& if\, {y}_{i}>0\end{array}\right.$$

The standard NB probability mass function, parameterized by its conditional mean ${\mu}_{i}$ and an overdispersion parameter $\phi$, is defined as:

$${P}_{NB}({Y}_{i}={y}_{i};{\mu}_{i},\phi )=\frac{\Gamma ({y}_{i}+1/\phi )}{{y}_{i}!\Gamma (1/\phi )}{\left(\frac{1}{1+\phi {\mu}_{i}}\right)}^{1/\phi }{\left(\frac{\phi {\mu}_{i}}{1+\phi {\mu}_{i}}\right)}^{{y}_{i}}$$

In the marginalized framework, the parameters are restructured to link covariates directly to the overall marginal mean (${\nu}_{i}={e}^{{X}_{i}^{^{\prime}}\alpha }$). As established in the manuscript, the relationship between the conditional mean of the count process (${\mu}_{i}$) and the marginal mean is expressed as:

$${\mu}_{i}={e}^{{X}_{i}^{^{\prime}}\alpha }(1+{e}^{{Z}_{i}^{^{\prime}}\lambda })$$

Thus, the full likelihood function for the mZINB model parameters $(\lambda ,\alpha ,\phi )$ is given below as indicated by the manuscript:

$$L(\lambda ,\alpha ,\phi |y)=\prod_{{y}_{i}}{\left(1+{e}^{{Z}_{i}^{^{\prime}}\lambda }\right)}^{-1}\prod_{{y}_{i}=0}\left[{e}^{{Z}_{i}^{^{\prime}}\lambda }+{\left(1+\phi {e}^{{X}_{i}^{^{\prime}}\alpha }(1+{e}^{{Z}_{i}^{^{\prime}}\lambda })\right)}^{-1/\phi }\right]\times \prod_{{y}_{i}>0}\left[\frac{\Gamma ({y}_{i}+1/\phi )}{{y}_{i}!\Gamma (1/\phi )}{\left(\frac{1}{1+\phi {e}^{{X}_{i}^{^{\prime}}\alpha }(1+{e}^{{Z}_{i}^{^{\prime}}\lambda })}\right)}^{1/\phi }{\left(\frac{\phi {e}^{{X}_{i}^{^{\prime}}\alpha }(1+{e}^{{Z}_{i}^{^{\prime}}\lambda })}{1+\phi {e}^{{X}_{i}^{^{\prime}}\alpha }(1+{e}^{{Z}_{i}^{^{\prime}}\lambda })}\right)}^{{y}_{i}}\right]$$

## Code availability

The *mzim* package developed to accompany this tutorial is available as open-source software via GitHub (https://github.com/cliattx/mzim). All data and analytic code to reproduce the empirical example presented in this manuscript are hosted on the Open Science Framework (OSF) at https://osf.io/82n79/overview?view_only=31617e58d54b4c18b81a39d83183f6d8.

## Declarations

### Open practices statement

The analysis code and the software package *mzim* described in this article are available at https://osf.io/82n79/overview?view_only=31617e58d54b4c18b81a39d83183f6d8. This study was not preregistered.
